# Differences in Physical Activity and Sedentary Behavior Patterns of Postmenopausal Women With Normal vs. Low Total Hip Bone Mineral Density

**DOI:** 10.3389/fspor.2020.00083

**Published:** 2020-07-09

**Authors:** Swati Chopra, Melissa M. Morrow, Che Ngufor, Emma Fortune

**Affiliations:** ^1^Leeds Institute of Rheumatic and Musculoskeletal Medicine, University of Leeds, Leeds, United Kingdom; ^2^Robert D. and Patricia E. Kern Center for the Science of Health Care Delivery, Department of Health Sciences Research, Mayo Clinic, Rochester, MN, United States; ^3^Division of Health Care Policy and Research, Department of Health Sciences Research, Mayo Clinic, Rochester, MN, United States; ^4^Division of Digital Health Sciences, Department of Health Sciences Research, Mayo Clinic, Rochester, MN, United States

**Keywords:** accelerometer, wearable sensors, bone mineral density, postmenopausal women, osteopenia

## Abstract

**Purpose:** Recent evidence suggests that sedentary behavior (SB) may be associated with bone health. This study compares free-living physical activity (PA) and SB distribution patterns of postmenopausal women with normal vs. low total hip bone mineral density (BMD).

**Methods:** Sixty nine post-menopausal women [mean (min-max) age: 61 (46–79) years] wore ActiGraph GT3X+ activity monitors on the bilateral ankles for 7 days in free-living. Participants were split into two groups: those with normal hip BMD (T-scores ≥-1.0; *N* = 34) and those with low hip BMD (T-scores <-1.0; *N* = 35) as defined by the World Health Organization. Daily active time, step counts, sedentary time, sedentary break number, and median sedentary bout length were estimated from ankle acceleration data. The distribution and accumulation patterns of time spent in sedentary bouts, sedentary breaks, and stepping bouts, and sedentary break and stepping bout lengths' variability were also investigated. Group differences were assessed using two-sampled *t*-tests and Mann-Whitney *U*-tests with significance levels of 0.5.

**Results:** Significant between group differences (*p* < 0.05) were in total daily active time [median (IQR): 257 (209–326) vs. 249 (199–299) min], step count [14,188 (10,938–18,646) vs. 13,204 (10,337–16,630) steps], sedentary time [669 (584–731) vs. 687 (615–753) min], and sedentary break number [93 (68–129) breaks vs. 88 (64–113) breaks], as well as median sedentary bout length [15.1 (11.9–22.1) vs. 15.8 (12.1–24.9) min]. Participants' sedentary bouts were found to be power law distributed with 52% of sedentary time occurring in bouts ≥20 min for the normal BMD group, and 58% for the low BMD group. Significant differences were observed between groups in sedentary bouts' and sedentary breaks' power distribution exponents (*p* < 0.0001) and patterns of sedentary and stepping time accumulation using the Gini index (*p* ≤ 0.0014). Variability was significantly lower for sedentary break and stepping bout lengths for the low BMD group (*p* ≤ 0.0001). Participants with lower hip BMD have longer sedentary bouts with shorter and less complex activity bouts compared to participants with normal hip BMD.

**Conclusion:** The results suggest healthier hip BMD may be associated with PA distributed more evenly throughout the day with shorter sedentary bouts. PA distribution should be considered in exercise-based bone health management programs.

## Introduction

Osteoporosis results in 1.5 million fractures (Gass and Dawson-Hughes, 2006) and a cost of nearly $20 billion in the U.S. each year (Burge et al., 2007), with the majority occurring in postmenopausal women (Khosla and Melton, 2007). In the U.S. alone, more than 44 million adults aged >50 years either have osteoporosis or are at high risk due to low bone mass (osteopenia) (Lim et al., 2009). The prevalence of osteoporosis increases from 19 to >50% in women aged 65–74 years and ≥85 years, respectively (Gass and Dawson-Hughes, 2006). Hip fractures account for the majority of the disability, morbidity, and financial burden of osteoporosis (Johnell, 1997), and are associated with increased mortality (Abrahamsen et al., 2009).

Osteopenia is considered as a precursor to osteoporosis if left untreated. Preventive treatments are recommended to people with osteopenia in order to delay or prevent bone loss progressing to osteoporosis. Convincing evidence indicates that physical activity (PA) is beneficial in the prevention of osteoporotic fractures and is one of the main modifiable risk factors (Kannus, 1999). Weight-bearing exercise, even with low impact forces on bone, is effective in maintaining bone mineral density (BMD) in postmenopausal women (Grove and Londeree, 1992). Studies on the effects of long reduced weight bearing periods, involving bed rest and time spent in reduced gravity, have suggested a resulting bone resorption increase (Zerwekh et al., 1998) and a decrease in the stimulation of bone formation (Zwart et al., 2007) due to the lack of PA. Conflicting results have been reported with some studies finding no effect of PA or SB on bone mass (Calderon-Garcia et al., 2013). However, this may be due to the measurement of bone density in other body regions such as the phalanges, and the subjective measurement of PA and SB using questionnaires which have been reported to give marked overestimations of PA (Troiano et al., 2008). A small number of recent studies have investigated the effects of objectively measured sedentary time on BMD (Chastin et al., 2014; Braun et al., 2017). Sedentary time and number of breaks in sedentary time, but not PA, were found to be significant predictors of osteoporosis or osteopenia at the femoral neck in postmenopausal women (Braun et al., 2017). Duration of sedentary bouts rather than frequency appeared to be detrimentally associated with total hip BMD in women ranging from 23 to 90 years old (Chastin et al., 2014). However, (Kozey-Keadle et al., 2011; Stansfield et al., 2015), the associations of more complex measures of PA or sedentary behavior (SB), such as sedentary or active time distribution, accumulation, and PA variability, with bone health measures have not yet been investigated for postmenopausal women. Recent studies have found significant differences in sedentary time distribution and accumulation pattern with no significant differences in total sedentary time between active and sedentary young to middle-aged adults (Chastin and Granat, 2010), and between individuals suffering from chronic lower back pain, chronic fatigue syndrome, Parkinson's disease, and controls (Chastin and Granat, 2010; Chastin et al., 2010). These more complex measures have also been applied to give more detailed analyses of the habitual PA patterns of individual's with Parkinson's Disease (Lord et al., 2011, 2013; Rochester et al., 2012; Hiorth et al., 2016).

An accelerometer-based algorithm to measure upright active time, and steps for gait velocities as low as 0.1 m/s was developed and validated (Fortune et al., 2014a, 2015; Lugade et al., 2014), and extended to measure free-living sedentary time distribution and accumulation patterns in a small cohort of postmenopausal women (Fortune et al., 2017). The current study's aim was to compare the free-living PA and SB, including distribution and accumulation patterns, of a larger cohort of postmenopausal women with low total hip BMD to those with normal total hip BMD in their home and community environments. We hypothesize that distribution, variability, and accumulation patterns of PA and SB may indicate differences in bone health, and that they should be considered in addition to overall PA and SB volume in bone health management.

## Methods And Materials

### Human Participants

Seventy post-menopausal women participated in this study, with a mean (SD) age of 61.3 (7.4) years with a range of 46–79 years, and a BMI of 26.1 (4.9) kg^.^m^−2^. Participants were recruited from previous and ongoing studies on bone health and through advertising in the local community. The full list of exclusion criteria are described in Madansingh et al. (2020) and included being self-reportedly post-menopausal for at least 1 year, <2 years of self-reported starting, stopping or modifying osteoporosis treatment, currently taking prescription medication which may cause BMD changes, induced menopause, use of an assistive walking device or any lower body amputations or orthotics, or a BMI of over 40 kg/m^2^. The protocol (IRB # 16-003202) was approved by the Institutional Review Board and participants provided written informed consent prior to participation.

### Data Collection

Accelerometer data were acquired as participants wore ActiGraph GT3X+ (ActiGraph LLC, Pensacola, FL, USA) activity monitors (AMs) on the bilateral ankles for seven consecutive days in their free-living environments. AMs were secured with straps around the ankle, located just above the lateral malleoli. Participants were instructed to wear the AMs at all times except during sleeping, bathing, or swimming. A valid AM day was defined as ≥10 wear hours per day (Troiano et al., 2008). Each axis was sampled at a rate of 100 Hz. Each participant's total hip BMD and corresponding T-score (BMD normalized by the young adult reference BMD value) were collected from their non-dominant hip within a month of their AM data collection. BMD was measured using a GE-Lunar iDXA Dual X-ray Absorptiometry (DXA) scanner (GE Healthcare, Madison, WI). The T-scores were calculated by the scanner software platform. Participants were split into two groups for analyses: (1) normal BMD (T-score ≥-1.0), and (2) low BMD (T-score <-1.0).

### Data and Statistical Analyses

The PA parameters of interest were: (1) daily active time, (2) daily step counts, (3) daily stepping bout length distribution (α_w_), (4) daily stepping time accumulation pattern (G_w_), and (5), daily variability in stepping bout time (S_2w_). SB parameters of interest were: (1) daily sedentary time, (2) daily number of breaks in sedentary time, (3) median daily sedentary bout length, (4) daily sedentary bout length distribution (α_sed_), (5) daily sedentary time accumulation pattern (G_sed_), (6) daily sedentary break length distribution (α_act_), (7) daily sedentary break accumulation pattern (G_act_), and (8) daily variability in sedentary break time (S_2act_). All accelerometer data post-processing and analysis were performed offline using MATLAB (Version 7.11.0, Mathworks, MA). Dynamic activity, steps and sedentary epochs were detected using algorithms previously developed and validated for younger to middle-aged participants with gait velocities ranging from 0.1 to 4.8 m^.^s^−1^ and during a simulated free-living protocol in the lab using Mayo Clinic accelerometer-based AMs (Fortune et al., 2014a; Lugade et al., 2014), and for older adult participants with gait velocities ranging from 0.5 (0.02) to 1.7 (0.06) m^.^s^−1^ using the ActiGraph GT3X+ AMs (Fortune et al., 2015, 2017). We additionally previously found that there were no significant differences between the daily step counts yielded by our step detection algorithm and the step counts yielded by the ActiLife low frequency extension step detection algorithm using the free-living data from the cohort of postmenopausal women also used in the current manuscript (Madansingh et al., 2020). Similar to Fortune et al. (2017), in this study, acceleration data were classified as either active (upright dynamic activity) or sedentary (static and/or lying down posture) on a second by second basis.

As described in detail in Fortune et al. (2014b), upright dynamic activity was identified for 1 s epochs when the vertical ankle angle estimation was <50 degrees, and the bodily motion component's signal magnitude area of the ankle acceleration exceeded 0.246 g or the acceleration data exceeded a scaling threshold of 1.5 within a range of 0.1–2.0 Hz when a continuous wavelet transform was applied. This algorithm was applied to each ankle AM and for every 1 s epoch where activity was detected, the epoch was defined as an activity segment. Step events were determined by applying an adaptive threshold peak detection algorithm to the ankle acceleration data for all upright dynamic activity periods for each limb (Fortune et al., 2015). Complete posture, activity, and step detection algorithm development and validation details can be found in our previous studies (Fortune et al., 2014a,b, 2015; Lugade et al., 2014).

**Figure 1:**
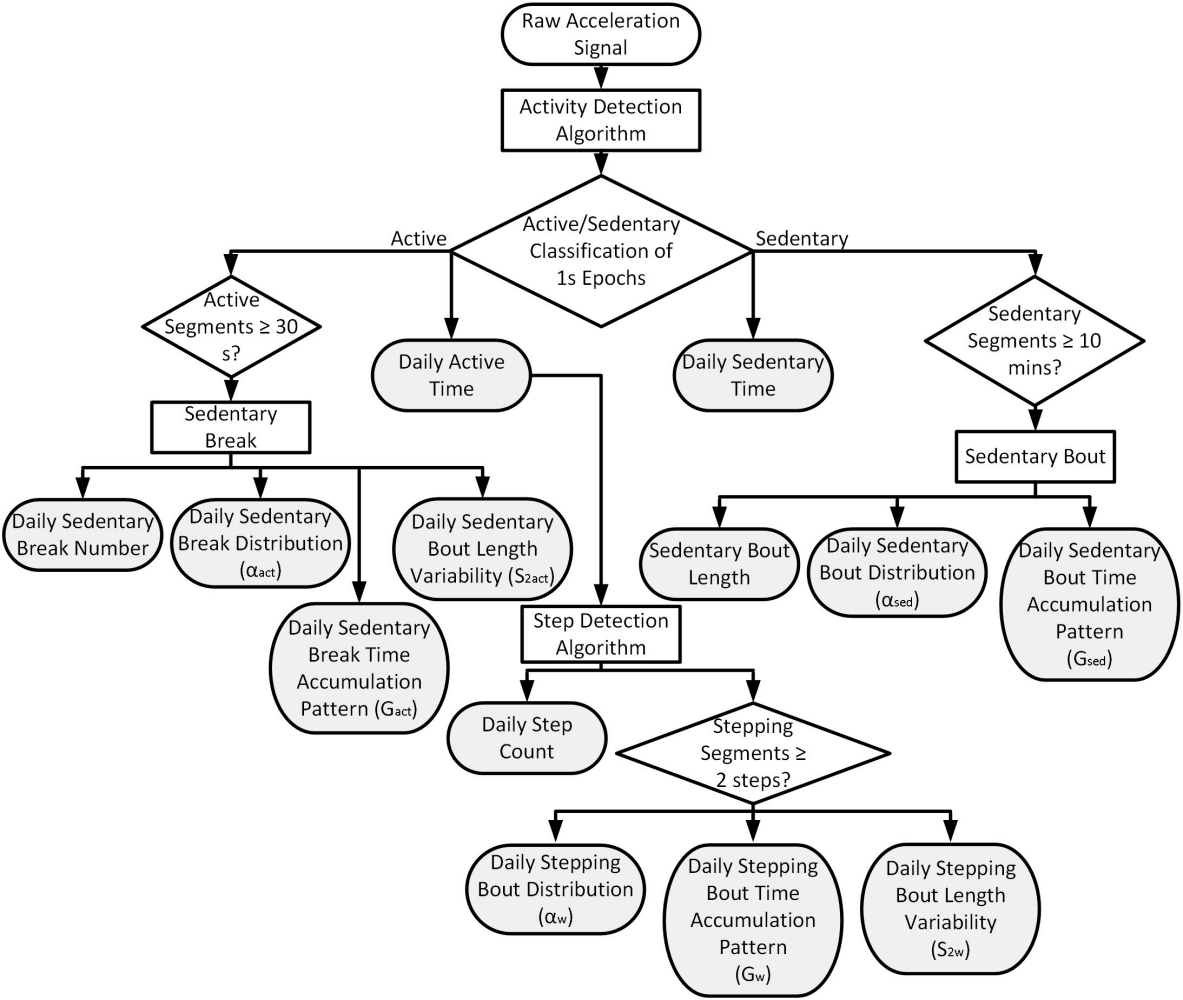
Schematic flowchart describing the algorithm steps. The filled-in gray blocks mark the 13 physical activity and sedentary behavior outcome measures.

Similar to Fortune et al. (2017), in this study, for every 1 s epoch where either activity was not detected or a laying down posture was detected, the epoch was defined as a sedentary segment. A sedentary break was identified when an activity segment was ≥30 s with at least 1 s of sedentary time preceding it (Fortune et al., 2017). A sedentary bout was defined as a segment where no activity was detected for at least 10 min (Chastin and Granat, 2010). A stepping bout was defined as any active segment where 2 or more steps occurred. A schematic flowchart of the algorithm steps is presented in Figure 1.

The relationship between sedentary bout length and density was investigated on a logarithmic scale to examine sedentary bout length distribution [7]. Exponent α was estimated from the histogram shape to characterize the sedentary bouts' power distribution, and quantify different SB strategies, with lower α exponents indicating the accumulation of sedentary time with larger percentages of long sedentary bouts. The pattern of sedentary time accumulation was described using the Lorenz curve and resulting Gini index (Chastin and Granat, 2010). The Gini index ranges from 0 (all sedentary bouts lengths contribute equally to sedentary time) to 1 (the longest sedentary bouts make up a very small percentage of total sedentary time). We additionally investigated sedentary break length and density, and the pattern of sedentary break time accumulation, as well as stepping bout length and density, and the pattern of stepping bout length using the power law characteristic exponent α and Gini index. For sedentary break or stepping bout lengths, lower α exponents indicate the accumulation of active time with larger percentages of long active bouts and a Gini index of 1 indicates the longest activity bouts make up a very small percentage of total active time. The variability, or within participant range, of sedentary break and stepping bout lengths, S_2act_ and S_2w_, was estimated as the standard deviation of the log normally distributed bout lengths via the maximum likelihood technique as described in Rochester et al. (2012). Higher variability values indicate a more varied pattern of walking. Shapiro-Wilk tests were used to test data for normality distribution. Students two-tailed *t*-tests and Mann-Whitney *U*-tests were used as appropriate to assess between group differences for all PA and SB parameters of interest. Significance level was set at 0.05.

## Results

Data from one participant were excluded from all analyses due to missing data as a result of one of the ActiGraph AMs ceasing to record data after only 1 day of AM wear. For the remaining 69 participants, we observed 100% compliance with all participants wearing their AMs for seven valid days. There were no significant differences between the groups' mean ages or BMIs (Table 1).

**Table 1:** Group demographic data including number of participants (*N*), and mean (min - max) age, BMI, BMD, and total hip T-scores.

**Group**	**Normal BMD**	**Low BMD**	***P*-Value**
*N*	34	35	-
Age (years)	60 (46–79)	63 (52–79)	0.07
BMI (kg/m^2^)	27 (18–40)	25 (20–34)	0.06
BMD (g/cm^2^)	0.99 (0.88–1.39)	0.79 (0.62–0.88)	-
T-Score	−0.2 (−1.0–3.0)	−1.8 (−3.1 to −1.1)	-

**Figure 2:**
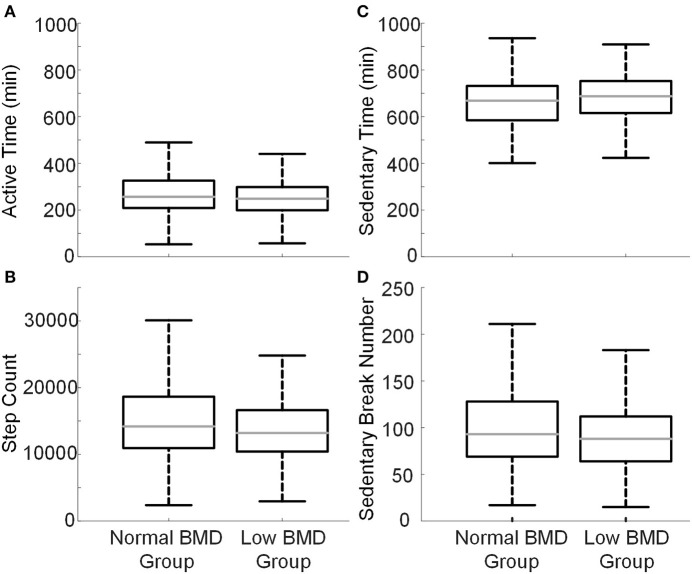
Boxplots representing daily **(A)** active time, **(B)** step counts, **(C)** sedentary time, and **(D)** sedentary break number for all participants in each group. The central line (gray) represents the median, the edges of the box are the 25th and 75th percentiles, and the error bars extend to ± 1.5 of the interquartile range from the median value.

Active time, step counts, and sedentary break number were significantly higher for the normal BMD group (Table 2, Figure 2). Sedentary time and sedentary bout length were significantly lower for the normal BMD group (Table 2, Figure 2). Participants in the normal BMD spent a mean (SD) of 70.32 (8.83) % of their time in SB, while participants in the low BMD group spent 71.88 (7.87) % of their time in SB. For the normal BMD group, 70 percent of sedentary bouts were <20 min but bouts >20 min contributed to 52% of total sedentary time. For the low BMD group, 64% of sedentary bouts were <20 min but bouts >20 min contributed to 58% of total sedentary time.

**Table 2:** Median (IQR) physical activity and sedentary behavior parameters across all participants in the normal and low bone mineral density (BMD) groups and *p*-values for between group differences.

**Parameter**	**Normal BMD**	**Low BMD**	***P*-Value**
Active time (mins)*	257 (209–326)	249 (199–299)	0.04
Step count*	14,188 (10938–18646)	13,204 (10337–16630)	0.01
Sedentary time (mins)*	669 (584–731)	687 (615–753)	0.02
Sedentary break number*	93 (68–129)	88 (64–113)	0.005
Sedentary bout length (mins)*	15.1 (11.9–22.1)	15.8 (12.1–24.9)	<0.0001
α_sed_*	2.6817 (2.6169–2.7822)	2.5556 (2.5341–2.6219)	<0.0001
σ_sed_*	0.0477 (0.0454–0.0661)	0.0403 (0.0358–0.0612)	-
G_sed_*	0.3326 (0.3205–0.3422)	0.3450 (0.3349–0.3496)	0.0014
α_act_*	3.0383 (3.0149–3.0630)	3.2214 (3.2010–3.2618)	<0.0001
σ_act_	0.0185 (0.0148–0.0258)	0.0206 (0.0172–0.0295)	-
G_act_	0.3685 (0.3558–0.3745)	0.3728 (0.3564–0.3832)	0.4977
S_2act_ (s)*	0.5487 (0.5401–0.5567)	0.5258 (0.5203–0.5293)	<0.0001
α_w_	1.7987 (1.7939–1.8014)	1.7932 (1.7889–1.8023)	0.7323
σ_w_	0.0021 (0.0017–0.0029)	0.0021 (0.0017–0.0029)	-
G_w_*	0.7264 (0.7150–0.7298)	0.7016 (0.6990–0.7073)	<0.0001
S_2w_ (s)*	1.2022 (1.1920–1.2061)	1.1900 (1.1857–1.1971)	0.0001

* *denotes a significant difference (p < 0.05)*.

**Figure 3:**
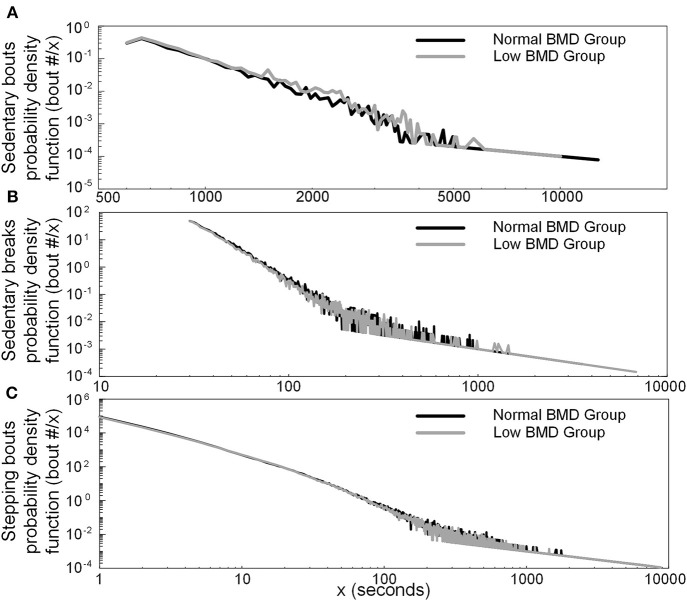
Distribution of **(A)** sedentary bout per sedentary bout length (x), **(B)** sedentary break per sedentary break length (x), and **(C)** stepping bout per stepping bout length (x) for all participants in each group.

**Figure 4:**
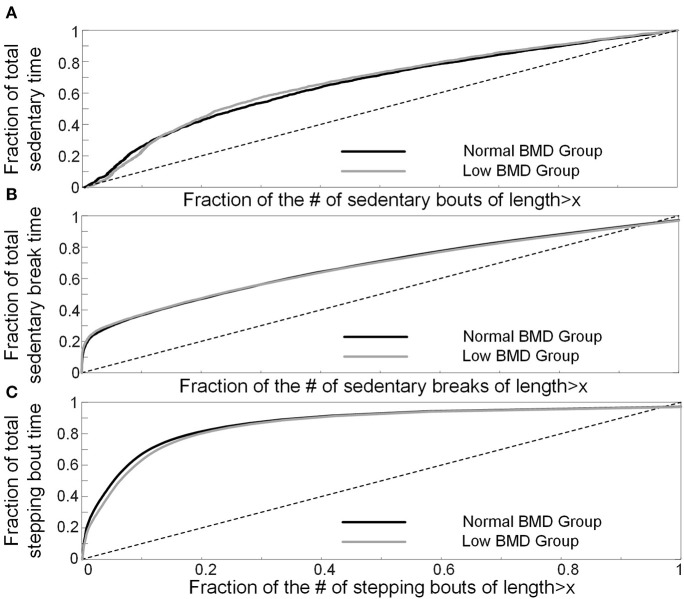
The Lorenz curves linking **(A)** the fraction of sedentary time to the proportion of sedentary bouts, **(B)** the fraction of sedentary break time to the proportion of sedentary break time, and **(C)** the fraction of stepping bout time to the proportion of stepping bout time for all participants in each group. The dashed line represents the line of perfect equality and the Gini index for each group is estimated as the area between the Lorenz curve and the line of perfect equality.

The sedentary bouts', sedentary breaks', and stepping bouts' distributions with respect to their lengths were similar to a power law distribution for both groups (Figure 3). There were significant differences between group differences in the power law characteristic exponent α for sedentary bouts and sedentary breaks but not for stepping bouts, with lower α values for sedentary bouts and higher α values for sedentary breaks for the low BMD group compared to the normal BMD group (Table 2, Figure 3). The standard errors on α for sedentary bouts, sedentary breaks, and stepping bouts (σ_sed_, σ_act_, σ_w_) were all <0.05 or <1.78% of α. There were significant between group differences in the Gini index for sedentary bouts and stepping periods but not sedentary breaks, with higher Gini index values for sedentary bouts and lower Gini index values for stepping periods for low BMD compared to normal BMD (Table 2, Figure 4). Variability in sedentary breaks and stepping bout lengths were significantly different between groups with higher variability in sedentary break and stepping bout lengths for the normal BMD group (Table 2, Figure 4).

## Discussion

The dose-response relationship between different PA and SB patterns and bone health remains to be established for post-menopausal women. As such, the goal of this study was to compare the free-living PA and SB distribution patterns of postmenopausal women with low hip BMD to those with normal hip BMD in their home and community environments.

The recommended daily step counts for adults to maintain a healthy life-style from government agencies around the world range from 8,500 to 10,000 steps per day (Tudor-Locke et al., 2011). Postmenopausal women have been reported to take between 3,528 and 19,958 steps per day (Kroemeke et al., 2014), similar to the step counts observed in the current study. Individuals taking ≥10,000 steps per day are defined as active and ≥12,500 steps per day as highly active (Tudor-Locke and Bassett, 2004). In the current study, the median daily step counts for both the normal and low hip BMD groups were slightly higher than the previous recommendation of 12,500 steps per day for postmenopausal women (Kroemeke et al., 2014).

Increased SB, independent of PA, is now considered as a public health risk (Harvey et al., 2015). Studies have shown the negative impact of SB on bone health, resulting in increased bone resorption with similar effects from frequent long continuous breaks of inactivity as that of prolonged bed-rest (Chastin et al., 2014). A recent longitudinal study has found an association between reduced bone loss with light intensity PA in older post-menopausal women, suggesting the addition of the breaking up of SB along with maintaining moderate-to-vigorous PA to help prevent osteoporosis in post-menopausal women (Rodríguez-Gómez et al., 2019).

The daily active and sedentary time estimates observed in the current study were similar to previous reports in the literature (Arnardottir et al., 2013; Wanner et al., 2013). The mean daily sedentary break numbers reported in this study were also comparable to previous studies (Bankoski et al., 2011; Fortune et al., 2017). Furthermore, participants in both groups spent a similar percentage (70 and 72%) of their day being sedentary which was also reported in previous studies on older adults (68–78%) (Arnardottir et al., 2013; Godfrey et al., 2013; Jefferis et al., 2015; Diaz et al., 2016; Fortune et al., 2017). The small differences in percentage of time spent sedentary between studies could be due to any number of factors such as algorithm differences, AM placement, sample sizes, participant age, or employment status.

A previous study found that daily sedentary time and sedentary break number, but not daily PA, were significant predictors of osteopenia or osteopenia at the femoral neck in postmenopausal women (Braun et al., 2017). Significant differences were observed in daily active time and step counts, as well as daily sedentary time and sedentary break numbers between groups in the current study. Consistent with our findings, in a study on women ranging from 23 to 90 years old, duration of sedentary bouts appeared to be negatively associated with total hip BMD (Chastin et al., 2014). It has been suggested that more complex SB pattern examination is needed when investigating the effects of SB (Esliger and Tremblay, 2007; Chastin and Granat, 2010; Healy et al., 2011). The scope of physical activity measurement has also been extended to include such more complex measures, with some evidence suggesting that they may be more sensitive measures in older adults than traditional daily volume measures such as daily active time or daily step count (Cavanaugh et al., 2007; Lord et al., 2011). In the current study, the power law characteristic distribution exponent of sedentary bout length was significantly lower in the group with low BMD compared to the group with normal BMD, indicating a larger proportion of the sedentary time being made up of long sedentary bouts. The significantly higher sedentary break length exponent for the low BMD group indicated a smaller proportion of active time being made of long activity bouts. The significantly higher Gini index of sedentary time accumulation and significantly lower Gini index of stepping time accumulation in the group with low BMD compared to the group with normal BMD in the current study mean that sedentary behavior was made up of longer sedentary bouts and physical activity was made up of shorter stepping bouts in the low BMD group compared to the normal BMD group. The lower variability in sedentary break and stepping bout lengths for the low BMD group suggests “less complex, less physiologically demanding patterns of activity” (Lord et al., 2011). In conclusion, the participants in the low BMD group break up their sedentary time, with shorter and less variable activity and stepping bouts, and into a smaller number of sedentary bouts of longer lengths, with a higher percentage of time spent in sedentary bouts longer than 20 min. The between group differences in sedentary bouts of longer lengths shown in our study are similar to those observed between healthy participants and participants with chronic conditions or diseases (Chastin and Granat, 2010; Chastin et al., 2010). The between group differences in physical activity volume, distribution, accumulation, and variability are also similar to those previously reported between healthy participants and individuals with Parkinson's Disease (Lord et al., 2013).

Causality cannot be inferred due to the cross-sectional nature of this study. While lower volumes of activity can lead to bone loss, the knowledge of having osteopenia or being at risk of developing osteoporosis can result in limited exercise participation due to fear of falling and the associated consequences (Resnick et al., 2014). PA and SB during earlier life, particularly during childhood and adolescence, result in much larger BMD changes compared to later life stages and can also be predictive of BMD in later life (Calderon-Garcia et al., 2013). Unfortunately, objective measurements from earlier life stages cannot be retrospectively obtained. Information on dosages for any participants undergoing osteoporosis treatment or prior history of taking prescription medication that may result in BMD changes may further explain between group differences but, unfortunately, was not recorded for this study. Due to the exclusion of individuals who use assistive walkers, the study cohorts are biased to exclude slow walkers and those with functional limitations reducing the generalizability of the study results. Future studies should investigate both the validity and applicability of these methods in postmenopausal women with lower functional levels. The participants included in this study were not age-matched between groups. However, no significant differences in age were detected between groups. Typically, AMs are worn on the hip (using accelerometry cut-points for sedentary vs. active classification Chastin et al., 2014) or thigh (incorporating postural detection to differentiate activity while sitting from activity while standing Chastin and Granat, 2010), while ankle placement has been shown to be optimal for step detection (Fortune et al., 2015; Korpan et al., 2015). We chose ankle placement for the current study as our primary goal was to investigate the effects of upright PA and dynamic loading (for which the ankle is the optimal location Fortune et al., 2014c) on hip BMD (Madansingh et al., 2020). As a thigh AM is needed to differentiate between standing and sitting postures, a limitation of the use of ankle AMs means that detected activity while sitting may be classified as PA rather than SB depending on the ankle orientation to the ground during sitting. Nonetheless, this is one of a few studies to investigate postmenopausal women's PA and SB distribution and accumulation patterns and their role in bone health and uses a novel algorithm with high accuracy for differentiating between stationary and low intensity movement.

Our data suggest that the distribution and accumulation of both PA and sedentary time, and PA variability may be important bone health management factors to consider in future studies. Our findings also highlight that the power law characteristic exponent appears to be an appropriate and sensitive measure to detect PA and SB differences between cohorts with normal and low hip BMD.

## Data Availability Statement

The datasets generated for this study are available on request to the corresponding author.

## Ethics Statement

The studies involving human participants were reviewed and approved by Mayo Clinic Institutional Review Board. The patients/participants provided their written informed consent to participate in this study.

## Author Contributions

EF directed the research. EF and SC drafted the article. MM and CN provided critical revisions. All authors have made substantial contributions to (1) the conception and design of the study, or acquisition of data, or analysis and interpretation of data, (2) drafting the article or revising it critically for important intellectual content, and (3) final approval of the version to be submitted. All authors contributed to the data analysis and interpretation of data and gave final approval of the version to be submitted.

## Conflict of Interest

The authors declare that the research was conducted in the absence of any commercial or financial relationships that could be construed as a potential conflict of interest.

## References

[B1] Abrahamsen B. van Staa T. Ariely R. Olson M. Cooper C. (2009). Excess mortality following hip fracture: a systematic epidemiological review. Osteoporos. Int. 20, 1633–1650. 10.1007/s00198-009-0920-3 19421703

[B2] Arnardottir N. Y. Koster A. Van Domelen D. R. Brychta R. J. Caserotti P. Eiriksdottir G.. (2013). Objective measurements of daily physical activity patterns and sedentary behaviour in older adults: age, gene/environment susceptibility-reykjavik study. Age Ageing 42, 222–229. 10.1093/ageing/afs160 23117467 PMC3575120

[B3] Bankoski A. Harris T. B. McClain J. J. Brychta R. J. Caserotti P. Chen K. Y.. (2011). Sedentary activity associated with metabolic syndrome independent of physical activity. Diabetes Care 34, 497–503. 10.2337/dc10-0987 21270206 PMC3024375

[B4] Braun S. I. Kim Y. Jetton A. E. Kang M. Morgan D. W. (2017). Sedentary behavior, physical activity, and bone health in postmenopausal women. J. Aging Phys. Act. 25, 173–181. 10.1123/japa.2016-0046 27620371

[B5] Burge R. Dawson-Hughes B. Solomon D. H. Wong J. B. King A. Tosteson A. (2007). Incidence and economic burden of osteoporosis-related fractures in the United States, 2005–2025. J. Bone Miner. Res. 22, 465–475. 10.1359/jbmr.061113 17144789

[B6] Calderon-Garcia J. F. Lavado-Garcia J. M. Martin R. R. Moran J. M. Canal-Macias M. L. Pedrera-Zamorano J. D. (2013). Bone ultrasound and physical activity in postmenopausal Spanish women. Biol. Res. Nurs. 15, 416–421. 10.1177/1099800412459800 22997347

[B7] Cavanaugh J. T. Coleman K. L. Gaines J. M. Laing L. Morey M. C. (2007). Using step activity monitoring to characterize ambulatory activity in community-dwelling older adults. J. Am. Geriatr. Soc. 55, 120–124. 10.1111/j.1532-5415.2006.00997.x 17233695

[B8] Chastin S. F. Baker K. Jones D. Burn D. Granat M. H. Rochester L. (2010). The pattern of habitual sedentary behavior is different in advanced Parkinson's disease. Mov. Disord. 25, 2114–2120. 10.1002/mds.23146 20721926

[B9] Chastin S. F. Granat M. H. (2010). Methods for objective measure, quantification and analysis of sedentary behaviour and inactivity. Gait Posture 31, 82–86. 10.1016/j.gaitpost.2009.09.002 19854651

[B10] Chastin S. F. Mandrichenko O. Helbostadt J. L. Skelton D. A. (2014). Associations between objectively-measured sedentary behaviour and physical activity with bone mineral density in adults and older adults, the NHANES study. Bone 64, 254–262. 10.1016/j.bone.2014.04.009 24735973

[B11] Diaz K. M. Howard V. J. Hutto B. Colabianchi N. Vena J. E. Blair S. N.. (2016). Patterns of sedentary behavior in US middle-age and older adults: the REGARDS study. Med. Sci. Sports Exerc. 48, 430–438. 10.1249/MSS.0000000000000792 26460633 PMC4760895

[B12] Esliger D. W. Tremblay M. S. (2007). Physical activity and inactivity profiling: the next generation. Appl. Physiol. Nutr. Metab. 32:S195–S207. 10.1139/H07-107 19377544

[B13] Fortune E. Lugade V. Amin S. Kaufman K. R. (2015). Step detection using multi-versus single tri-axial accelerometer-based systems. Physiol. Meas. 36:2519–2535. 10.1088/0967-3334/36/12/2519 26595421 PMC4838513

[B14] Fortune E. Lugade V. Morrow M. Kaufman K. (2014a). Validity of using tri-axial accelerometers to measure human movement–part II: step counts at a wide range of gait velocities. Med. Eng. Phys. 36, 659–669. 10.1016/j.medengphy.2014.02.006 24656871 PMC4030415

[B15] Fortune E. Lugade V. A. Kaufman K. R. (2014b). Posture and movement classification: the comparison of tri-axial accelerometer numbers and anatomical placement. J. Biomech. Eng. 136:051003. 10.1115/1.4026230 24337255 PMC4023813

[B16] Fortune E. Morrow M. M. Kaufman K. R. (2014c). Assessment of gait kinetics using triaxial accelerometers. J. Appl. Biomech. 30, 668–674. 10.1123/jab.2014-0037 25010675 PMC4332389

[B17] Fortune E. Mundell B. Amin S. Kaufman K. (2017). A pilot study of physical activity and sedentary behavior distribution patterns in older women. Gait Posture 57, 74–79. 10.1016/j.gaitpost.2017.05.014 28578137 PMC5865394

[B18] Gass M. Dawson-Hughes B. (2006). Preventing osteoporosis-related fractures: an overview. Am. J. Med. 119, S3–S11. 10.1016/j.amjmed.2005.12.017 16563939

[B19] Godfrey A. Lord S. Galna B. Mathers J. C. Burn D. J. Rochester L. (2013). The association between retirement and age on physical activity in older adults. Age Ageing 43, 386–393. 10.1093/ageing/aft168 24171945

[B20] Grove K. A. Londeree B. R. (1992). Bone density in postmenopausal women: high impact vs low impact exercise. Med. Sci. Sports Exerc. 24, 1190–1194. 10.1249/00005768-199211000-00002 1435170

[B21] Harvey J. A. Chastin S. F. Skelton D. A. (2015). How sedentary are older people? A systematic review of the amount of sedentary behavior. J. Aging Phys. Act. 23, 471–487. 10.1123/japa.2014-0164 25387160

[B22] Healy G. N. Matthews C. E. Dunstan D. W. Winkler E. A. Owen N. (2011). Sedentary time and cardio-metabolic biomarkers in US adults: NHANES 2003–06. Eur. Heart J. 32, 590–597. 10.1093/eurheartj/ehq451 21224291 PMC3634159

[B23] Hiorth Y. H. Larsen J. P. Lode K. Tysnes O. B. Godfrey A. Lord S.. (2016). Impact of falls on physical activity in people with Parkinson's disease. J. Parkinsons Dis. 6, 175–182. 10.3233/JPD-150640 26639446

[B24] Jefferis B. J. Sartini C. Shiroma E. Whincup P. H. Wannamethee S. G. Lee I.-M. (2015). Duration and breaks in sedentary behaviour: accelerometer data from 1566 community-dwelling older men (british regional heart study). Br. J. Sports Med. 49, 1591–1594. 10.1136/bjsports-2014-093514 25232029 PMC4363289

[B25] Johnell O. (1997). The socioeconomic burden of fractures: today and in the 21st century. Am J Med. 103, 20S−25S; discussion 25S−26S. 10.1016/S0002-9343(97)90023-1 9302894

[B26] Kannus P. (1999). Preventing osteoporosis, falls, and fractures among elderly people. Promotion of lifelong physical activity is essential. BMJ 318, 205–206. 10.1136/bmj.318.7178.205 9915707 PMC1114702

[B27] Khosla S. Melton L. III. (2007). Clinical practice. Osteopenia. N. Engl. J. Med. 356, 2293–2300. 10.1056/NEJMcp070341 17538088

[B28] Korpan S. M. Schafer J. L. Wilson K. C. Webber S. C. (2015). Effect of actigraph GT3X+ position and algorithm choice on step count accuracy in older adults. J. Aging Phys. Act. 23, 377–382. 10.1123/japa.2014-0033 25102469

[B29] Kozey-Keadle S. Libertine A. Lyden K. Staudenmayer J. Freedson P. S. (2011). Validation of wearable monitors for assessing sedentary behavior. Med. Sci. Sports Exerc. 43, 1561–1567. 10.1249/MSS.0b013e31820ce174 21233777

[B30] Kroemeke A. Zajac-Gawlak I. Pośpiech D. Gába A. Pridalov,á M. Pelclová J. (2014). Postmenopausal obesity: 12,500 steps per day as a remedy? Relationships between body composition and daily steps in postmenopausal women. Prz. Menopauzalny. 13, 227–232. 10.5114/pm.2014.44998 26327859 PMC4520368

[B31] Lim L. S. Hoeksema L. J. Sherin K. (2009). Screening for osteoporosis in the adult U.S. population: ACPM position statement on preventive practice. Am. J. Prev. Med. 36, 366–375. 10.1016/j.amepre.2009.01.013 19285200

[B32] Lord S. Chastin S. F. McInnes L. Little L. Briggs P. Rochester L. (2011). Exploring patterns of daily physical and sedentary behaviour in community-dwelling older adults. Age Ageing 40, 205–210. 10.1093/ageing/afq166 21239410

[B33] Lord S. Godfrey A. Galna B. Mhiripiri D. Burn D. Rochester L. (2013). Ambulatory activity in incident Parkinson's: more than meets the eye? J. Neurol. 260, 2964–2972. 10.1007/s00415-013-7037-5 23900754

[B34] Lugade V. Fortune E. Morrow M. Kaufman K. (2014). Validity of using tri-axial accelerometers to measure human movement—part I: posture and movement detection. Med. Eng. Phys. 36, 169–176. 10.1016/j.medengphy.2013.06.005 23899533 PMC3866210

[B35] Madansingh S. I. Ngufor C. G. Fortune E. (2020). Quality over quantity: skeletal loading intensity plays a key role in understanding the relationship between physical activity and bone density in postmenopausal women. Menopause 27, 444–449. 10.1097/GME.0000000000001486 31895180 PMC7108051

[B36] Resnick B. Nahm E. S. Zhu S. Brown C. An M. Park B.. (2014). The impact of osteoporosis, falls, fear of falling, and efficacy expectations on exercise among community-dwelling older adults. Orthop. Nurs. 33, 277–288. 10.1097/NOR.0000000000000084 25233207 PMC4170528

[B37] Rochester L. Chastin S. F. Lord S. Baker K. Burn D. J. (2012). Understanding the impact of deep brain stimulation on ambulatory activity in advanced Parkinson's disease. J. Neurol. 259, 1081–1086. 10.1007/s00415-011-6301-9 22086738

[B38] Rodríguez-Gómez I. Mañas A. Losa-Reyna J. Rodríguez-Mañas L. Chastin S. F. Alegre L. M.. (2019). Compositional influence of movement behaviours on bone health during ageing. Med. Sci. Sports Exerc. 51, 1736–1744. 10.1249/MSS.0000000000001972 30829961

[B39] Stansfield B. Hajarnis M. Sudarshan R. (2015). Characteristics of very slow stepping in healthy adults and validity of the activPAL3™ activity monitor in detecting these steps. Med. Eng. Phys. 37, 42–47. 10.1016/j.medengphy.2014.10.003 25455167

[B40] Troiano R. P. Berrigan D. Dodd K. W. Masse L. C. Tilert T. McDowell M. (2008). Physical activity in the United States measured by accelerometer. Med. Sci. Sports Exerc. 40, 181–188. 10.1249/mss.0b013e31815a51b3 18091006

[B41] Tudor-Locke C. Bassett D. R. (2004). How many steps/day are enough? Sports Med. 34, 1–8. 10.2165/00007256-200434010-00001 14715035

[B42] Tudor-Locke C. Craig C. L. Brown W. J. Clemes S. A. De Cocker K. Giles-Corti B. (2011). How many steps/day are enough? For adults. Int. J Behav. Nutr. Phys. Act. 8:79 10.1186/1479-5868-8-79 21798015 PMC3197470

[B43] Wanner M. Martin B. W. Meier F. Probst-Hensch N. Kriemler S. (2013). Effects of filter choice in GT3X accelerometer assessments of free-living activity. Med. Sci. Sports Exerc. 45, 170–177. 10.1249/MSS.0b013e31826c2cf1 22895373

[B44] Zerwekh J. E. Ruml L. A. Gottschalk F. Pak C. Y. (1998). The effects of twelve weeks of bed rest on bone histology, biochemical markers of bone turnover, and calcium homeostasis in eleven normal subjects. J. Bone. Min. Res. 13, 1594–1601. 10.1359/jbmr.1998.13.10.1594 9783548

[B45] Zwart S. R. Hargens A. R. Lee S. M. C. Macias B. R. Watenpaugh D. E. Tse K.. (2007). Lower body negative pressure treadmill exercise as a countermeasure for bed rest-induced bone loss in female identical twins. Bone 40, 529–537. 10.1016/j.bone.2006.09.014 17070743 PMC1876821

